# RNA-seq analysis of gene expression profiles in posttraumatic stress disorder, Parkinson’s disease and schizophrenia identifies roles for common and distinct biological pathways

**DOI:** 10.1007/s44192-022-00009-y

**Published:** 2022-03-03

**Authors:** Sian M. J. Hemmings, Patricia Swart, Jacqueline S. Womersely, Ellen S. Ovenden, Leigh L. van den Heuvel, Nathaniel W. McGregor, Stuart Meier, Soraya Bardien, Shameemah Abrahams, Gerard Tromp, Robin Emsley, Jonathan Carr, Soraya Seedat

**Affiliations:** 1 Department of Psychiatry, Faculty of Medicine and Health Sciences, Stellenbosch University, PO Box 241, Cape Town, 8000 South Africa; 2 South African Medical Research Council/Stellenbosch University Genomics of Brain Disorders Research Unit, Stellenbosch University, Cape Town, South Africa; 3 Systems Genetics Working Group, Department of Genetics, Stellenbosch University, Stellenbosch, South Africa; 4 Division of Molecular Biology and Human Genetics, Department of Biomedical Sciences, Stellenbosch University, Cape Town, South Africa; 5 DSI–NRF Centre of Excellence for Biomedical Tuberculosis Research, Stellenbosch University, Cape Town, South Africa; 6 South African Medical Research Council Centre for Tuberculosis Research, Stellenbosch University, Cape Town, South Africa; 7 South African Tuberculosis Bioinformatics Initiative, Stellenbosch University, Cape Town, South Africa; 8 Centre for Bioinformatics and Computational Biology, Stellenbosch University, Stellenbosch, South Africa; 9 Division of Neurology, Department of Medicine, Faculty of Medicine and Health Sciences, Stellenbosch University, Cape Town, South Africa

## Abstract

Evidence suggests that shared pathophysiological mechanisms in neuropsychiatric disorders (NPDs) may contribute to risk and resilience. We used single-gene and network-level transcriptomic approaches to investigate shared and disorder-specific processes underlying posttraumatic stress disorder (PTSD), Parkinson’s disease (PD) and schizophrenia in a South African sample. RNA-seq was performed on blood obtained from cases and controls from each cohort. Gene expression and weighted gene correlation network analyses (WGCNA) were performed using *DESeq2* and *CEMiTool,* respectively. Significant differences in gene expression were limited to the PTSD cohort. However, WGCNA implicated, amongst others, ribosomal expression, inflammation and ubiquitination as key players in the NPDs under investigation. Differential expression in ribosomal-related pathways was observed in the PTSD and PD cohorts, and focal adhesion and extracellular matrix pathways were implicated in PD and schizophrenia. We propose that, despite different phenotypic presentations, core transdiagnostic mechanisms may play important roles in the molecular aetiology of NPDs.

**Supplementary Information:**

The online version contains supplementary material available at 10.1007/s44192-022-00009-y.

## Introduction

Psychiatric disorders, such as posttraumatic stress disorder (PTSD) and schizophrenia, and neurodegenerative disorders, such as Parkinson’s disease (PD), pose an immense burden to society [1, 2]. PTSD is a stress-related disorder that occurs consequent to an identifiable proximate traumatic event. It has a prevalence of approximately 8% in the general population [3]. The disorder is particularly relevant in South Africa, given the high trauma burden, as reported by the South African Stress and Health Study [4]. Schizophrenia is a severe psychiatric disorder that affects almost 1% of the world’s population [5]. The illness typically has an onset in early adulthood and patients frequently experience multiple psychotic relapses and admissions to hospital, enduring deficits in cognitive, social and occupational functioning, and a significantly decreased life expectancy [6–8]. PD is a progressive, debilitating disease with substantial physical, psychological and social implications, and is the second most common neurodegenerative disorder after Alzheimer’s disease (AD). The global age-standardised rate for PD is 33 per 100,000 people, adjusted for disability life-years or DALYs [9].

PTSD, schizophrenia and PD are all multifactorial disorders, with a combination of genetic and environmental factors contributing substantially to their aetiology [10–12]. However, despite recent advances in biomarker and genetics studies, the pathophysiology of both PTSD and schizophrenia remains poorly understood. Likewise, although pathologically characterised by loss of nigrostriatal dopaminergic innervation, PD is considered an idiopathic disorder with Mendelian inheritance occurring in only 3−5% of patients [13].

Shared aetiologies may underlie these neuropsychiatric disorders (NPDs). For example, in a recent genome-wide association study (GWAS), it was found that *PRKN*, a PD-associated gene involved in proteasome-dependent degradation of proteins, was found to be associated with PTSD in a European population [14]. This finding was supported recently by research from our group [15]. Indeed, accumulating evidence indicates that PTSD is associated with increased risk for neurodegenerative disorders, such as PD and AD [16–18]. In addition, individuals who develop adjustment disorder as a result of inability to cope with stress have been found to be more likely to develop PD later in life [19]. Studies have also shown that stress can induce neurotoxicity in brain regions such as the substantia nigra, prefrontal cortex and hippocampus, resulting in neurodegenerative pathological alterations [20].

The association between schizophrenia and PD is evident on a clinical as well as molecular level. Parkinson-like motor abnormalities, independent of antipsychotic treatment, have been observed in patients with schizophrenia spectrum disorders [21–24] and PD patients often exhibit pathological features that overlap with schizophrenia, including hallucinations, psychosis and cognitive dysfunction [25–28]. In addition, a recent GWAS investigation identified nine loci that were jointly associated with both PD and schizophrenia [29], with seven of these loci common in previous studies [30–34]. Deletions in 22q11 have been found to be a strong genetic risk factor for schizophrenia [35] and a number of studies suggest that individuals with 22q11 deletions also develop early-onset PD [36–39]. On a molecular level, alterations in dopaminergic neurotransmission play a central role in both disorders—PD is associated with loss of dopaminergic neurons of the substantia nigra pars compacta, whereas schizophrenia has been found to be associated with hypodopaminergic activity in the prefrontal cortex and hyperactive dopaminergic activity in the mesolimbic and striatal regions of the brain [40, 41].

Prevalence estimates of PTSD in schizophrenia range from 12 to 29%, which is higher than that for the general population [42–44]. Psychotic symptoms typical of schizophrenia have been found to occur with a higher frequency than expected in PTSD patients [45–47]. In addition, symptoms of depersonalisation and derealisation, which form a dissociative subtype of PTSD, are also exhibited by patients with schizophrenia [48]. Although there is limited research into the genetic overlap between the two disorders, polygenic risk score analysis indicates a genetic overlap between PTSD and schizophrenia [49].

Due to converging lines of genetic, molecular, neurobiological and phenotypic evidence supporting shared pathophysiology underlying PTSD, PD and schizophrenia, we aimed to identify unique as well as shared pathways underlying these three disorders. We employed RNA-seq whole-transcriptome analysis, followed by analysis of genes that are co-expressed within modular networks identified within each disease cohort, using weighted gene correlation network analysis (WGCNA). WGCNA is a co-expression analysis that clusters genes with highly correlated expression levels into numerous modules, each module comprising genes with similar expression patterns. This facilitates our understanding of how genes within the modules may interact with one another, and may shed light on biological mechanisms underlying the disorders in question, as genes with similar function will usually exhibit strong correlations in expression [50, 51]. Highly-connected genes within modules, known as “hub genes”, are often important to the functionality of the module [52], and as such, may play a role in the aetiology of the disorder. Compared to analyses at the single-gene level, WGCNA represents an integrated, network-level approach, enabling the identification of altered gene co-expression within modules, and permits identification of broad pathological mechanisms underlying PTSD, PD and schizophrenia, even if the contributing genes are disorder-specific.

## Methods

The three diagnostic cohorts were recruited as part of the Shared Roots Study, conducted in Cape Town, South Africa. The study was approved by Stellenbosch University’s Health Research Ethics Committee (Ethics Approval Number: N13/08/115). Participants were recruited through purposive sampling at Stellenbosch University Faculty of Medicine and Health Sciences (Tygerberg Campus) and at Stikland Hospital, in Cape Town. Inclusion criteria for all participants was that they were willing and able to provide informed consent; were 18 years and older; all self-identified as being part of the South African Coloured population (as termed in the South African census); and were able to read and write Afrikaans or English. Participants were excluded if they did not provide informed consent; were pregnant or were younger than 18 years of age.

### Demographic and clinical assessment

A sociodemographic questionnaire was administered to gather data including age, sex, education, employment, income, marital status and ethnicity. A participant was considered a smoker if he/she had ever smoked cigarettes in their lifetime. Likewise, a participant was considered to have consumed alcohol if he/she had done so in their lifetime. We did not quantify alcohol consumption for this analysis.

The main criterion separating cases and controls in each of the cohorts rested on the clinical diagnosis of the disorder under investigation in each cohort–PTSD, schizophrenia or Parkinson’s Disease. All other inclusion and exclusion criteria are the same for cases and controls within each cohort.

Diagnosis of PTSD was made according to Diagnostic and Statistical Manual of Mental Disorders (DSM–5) [53] criteria with the Clinician-Administered Posttraumatic Stress Disorder Scale for DSM-5 (CAPS-5) [54]. All controls in the PTSD cohort were trauma-exposed (based on DSM-5 criteria). Schizophrenia was diagnosed using the Structured Clinical Interview for DSM-IV (SCID) [55]. A diagnosis of PD was ascertained by a neurologist, according to UK Brain Bank Criteria [56].

Metabolic syndrome (MetS), which represents a cluster of risk factors for cardiovascular disease and type II diabetes, was assessed as an objective in the parent study. MetS has been found to significantly alter gene expression profiles [57] and was therefore considered as a covariate in the current study. Utilising the harmonised Joint Interim Statement (JIS) criteria [58], the presence of any three of the following five risk factors indicated a positive MetS diagnosis: (i) raised waist circumference (≥ 90 cm [59]); (ii) raised triglycerides (> 1.7 mmol/l); (iii) low HDL-C (men < 1.0 mmol/l, women < 1.3 mmol/l); (iv) raised blood pressure ≥ 130/85 mmHg or on hypertension treatment; and (v) raised fasting glucose ≥ 5.6 mmol/l or on diabetes treatment.

Childhood trauma was assessed using the Childhood Trauma Questionnaire (CTQ) [60]. Based on CTQ total score, the following cut-offs have been suggested: none to minimal trauma (25–36), low to moderate trauma (41–51), moderate to severe trauma (56–68) and severe to extreme trauma (73–125) [60]. We thus used a score ≥ 41 to identify individuals as having childhood trauma, as this represents the lowest level score indicative of abuse or neglect for each subscale. Major Depressive Disorder (MDD) was assessed using the MINI International Neuropsychiatric Interview, version 6.0 (MINI) [61]. Participants with current or lifetime MDD were classified as having MDD.

### RNA isolation

Blood samples were collected from all participants using PAXgene Blood Collection tubes (Preanalytix, Switzerland), and RNA isolation was done using the PAXgene Blood RNA Kit (Qiagen, CA, USA). Samples were assessed for an RNA integrity number (RIN) using the Agilent 2100 Bioanalyzer (Agilent Technologies, California, USA). A RIN value of ≥ 7 is generally regarded as good quality, intact RNA [62].

### RNA sequencing and analysis

#### Library preparation and sequencing

RNA-sequencing (RNA-seq) was performed at The Kinghorn Centre for Clinical Genomics (KCCG), Australia. Illumina’s TruSeq Stranded Total RNA Library Prep Kit with Ribo-Zero Gold High Throughput was used for library preparation. Synthetic spike-ins (Sequins) were added at the point of the initial RNA dilution (1:50 dilution). Adapter dimers were detected in final library trace of the schizophrenia and PD samples, and all samples were therefore subjected to second bead clean-up, using a bead:sample ratio of 0.9. Following the bead clean-up, 14 samples (5 PD and 9 schizophrenia samples) still exhibited adapter dimers, and another bead clean-up procedure, using a bead:sample ratio of 0.85, was performed, after which the adapter dimers were removed. Since the differential treatment of the samples may introduce batch effects, we included the additional bead clean-up as a covariate in the investigations of the PD and schizophrenia cohorts.

Each sample was uniquely indexed, pooled and subsequently sequenced across ten lanes to minimise the potential for batch effects. Paired-end sequencing was performed on the Illumina HiSeq 2500 sequencing system, with a read length of 125 base pairs (bp) and sequencing depth of 50 million paired-end reads per sample.

### RNA-seq mapping

The quality of the raw reads was assessed using FastQC tool (Version 0.1 1.5), and adaptor-end trimming was conducted using Trim Galore! (Version 0.4.2). Alignment of the RNA-seq reads to the Ensembl human reference genome (hg38/GRCH38) was performed using Spliced Transcripts Alignment to a Reference (STAR; Version 2.6.0c) [63]. The *quantMode GeneCounts* option was selected to generate raw gene-wise read counts for each sample.

### Statistical analysis

The distributions of quantitative data were tested for normality using the Shapiro–Wilk test. Normal and asymmetrical data were summarised as mean and standard deviation, or median and interquartile range, respectively. Qualitative data were summarised as counts. To control for the effect of blood cell type composition variability on gene expression, blood cell type proportions were estimated using the ‘*immunoStates*’ expression matrix [64], and the *DeconRNASeq* package (317 genes across 20 cell types) [65] in R [66]. Cell types with the greatest composition variability have the potential to confound the gene expression results. Variables that exhibited significantly different distributions or frequencies between cases and controls in each diagnostic group were included as covariates in the gene expression analyses.

Exploratory analysis of the RNA-seq data was conducted using the R package *DaMIR* [67]. The *DaMiR*.*sampleFilt* function assesses the mean absolute correlation of each sample and removes those samples with a correlation lower than the value set in the *th.corr* argument. This was set at 0.9 in the current study.

We used the R package *variancePartition* [68] to assess the degree to which selected clinical and technical variables might influence gene expression and potentially confound the analysis. The package implements a linear mixed model method to characterise the contribution of selected variables to transcriptional variability. In the present study, total variance in gene expression was partitioned into the variance attributable to RIN, age, sex, nicotine use, and neutrophil and CD16 monocyte cell proportions in each cohort. Metabolic syndrome was included as a variable in the PTSD and PD cohorts, but not in the schizophrenia cohort (due to low numbers of MetS), and the additional bead clean-up was included as a variable in the PD and schizophrenia cohorts. Continuous variables (age, RIN, and neutrophil and CD16 monocyte proportions) were modelled as fixed effects, while categorical variables were modelled as random effects.

Differential expression analysis was performed using *DESeq2* [69], which analyses differences in expression based on a negative binomial generalised linear model, adjusting for covariables as necessary. Statistical significance was assessed via the Wald test. The false discovery rate (FDR) was controlled for at 10% using the Benjamini–Hochberg method [70] for each pairwise comparison. Neutrophil and CD16 monocyte proportions, age, sex, RIN, and smoking status were included as covariates in all models. Metabolic syndrome was included as a covariate in the PTSD and PD models, but not the schizophrenia model, given the low frequency of MetS diagnosed in that cohort (12.5% and 5.6% in cases and controls, respectively (Table 1)). Current medication for hypertension was also included as a covariate in the PD cohort.

**Table 1:** Clinical, demographic and haematological information for selected variables in the PTSD, schizophrenia and PD cohorts

Demographic/clinical variable	PTSD (n = 79)			PD^a^ (n = 30)			Schizophrenia^a^ (n = 34)		
	Case	Control	*p*	Case	Control	*p*	Case	Control	*p*
	(n = 40)	(n = 39)		(n = 12)	(n = 18)		(n = 16)	(n = 18)	
Age (years) (mean, SD)	44.13 (9.38)	45.32 (10.40)	0.595	63.52 (7.29)	61.93 (6.45)	0.505	24.18 (4.79)	23.4 (3.13)	0.458
Sex (Female, %)	33 (82.5)	31 (79.5)	0.733	5 (41.7)	7 (38.9)	0.879	8 (50.0)	8 (44.4)	0.746
Education (n, %)									
Grade 6 and below	2 (5.0)	0 (0)	0.414^d^	1 (8.3)	0 (0)	0.443^d^	0 (0)	0 (0)	0.711^d^
Grades 7–9	12 (30.0)	17 (43.6)		5 (41.7)	8 (44.4)		5 (31.3)	3 (16.7)	
Grades 10–12	24 (60.0)	20 (51.3)		4 (33.3)	9 (50.0)		9 (56.3)	11 (61.1)	
Tertiary	2 (5.0)	2 (5.1)		2 (16.7)	1 (5.6)		2 (12.5)	4 (22.2)	
Metabolic syndrome (yes, %)	20 (50.0	19 (48.7)	0.733	7 (58.3)	10 (55.6)	0.880	2 (12.5)	1 (5.6)	0.591^d^
Childhood trauma (yes, %)	37 (92.5)	24 (61.5)	**0.001**^b^	1 (8.3)	4 (22.2)	0.622^d^	8 (50.0)	12 (66.7)	0.324
CTQ score (median, IQR)	51.5 (45.75–71.25)	41 (32.5–56)	**0.002**^c^	32.5 (30.5–35.5)	34.5 (30.25–39.75)	0.337^c^	40.5 (38.5–48.25)	44.5 (40–57)	0.276^c^
MDD (yes, %)	24 (60.0)	9 (23.1)	**0.001**^b^	0 (0)	2 (11.1)	0.503	3 (18.8)	1 5.6)	0.323^d^
Smoking (yes, %)	25 (62.5)	21 (53.8)	0.436	5 (41.7)	13 (72.2)	0.094	12 (75.0)	7 (38.9)	**0.034**
Alcohol (yes, %)	32 (80.0)	28 (71.8%)	0.394	8 (66.7)	15 (83.3)	0.392^d^	11 (68.8)	11 (61.1)	0.642
Currently on anti-hypertensives (yes, %)	10 (25.0)	12 (30.8)	0.567	8 (66.7)	5 (27.8)	0.061^d^	0 (0%)	0 (0)	1
CD16 Monocyte^e^	0.22 (0.08)	0.23 (0.09)	0.542	0.24 (0.11)	0.28 (0.10)	0.340	0.33 (0.07)	0.29 (0.09)	0.189
Neutrophil (median, IQR) ^e^	0.36 (0.31–0.41)	0.39 (0.32–0.42)	0.202	0.348 (0.24–0.41)	0.3065 (0.23–0.35)	0.79^c^	0.37 (0.26–0.42)	0.32 (0.25–0.39)	0.334
RIN (medin, IQR)	8.8 (8.4–9.0)	8.8 (8.4–9.1)	0.637^c^	7.45 (6.95–8.1)	7.6 (7.0–8.1)	0.82^c^	7.9 (7.7–8.2)	7.8 (7.2–8.2)	0.436^c^

^a^Excluding outliers; ^b^Mann-Whitney U test; ^c^Kruskal-Wallis;^d^Fisher's exact test; ^e^estimated proportions; significant p-values (p < 0.05) indicated in bold

Weighted gene co-expression analysis was performed using the R package Co-Expression Modules identification Tool (*CEMiTool*) [71]. We applied the *CEMiTool* *vst()* function, which applies an arcsin-based variance stabilizing transformation if the correlation between the means and variances of genes is greater than 0.5 (vst) and also corrected for covariates included in the gene expression models for each NPD analysis using the *empiricalBayesLM* function implemented in the WGCNA package [72] prior to data input into *CEMiTool*.

As a first step, *CEMiTool* implements an unsupervised gene filtering step, based on the inverse gamma distribution. All default parameters were applied, with a variance filter p-value of 0.1. The beta-value (β), which represents the soft-thresholding power required to construct network adjacency, was selected using the default *CEMiTool* algorithm. Network construction was done using the unsigned method; in other words, up- and down regulated genes may be located in the same modules. We assume, therefore, that genes with a strong unsigned correlation are co-regulated. Gene-set enrichment analysis (GSEA) was performed using *fgsea* [73]*,* implemented in *CEMiTool*, in order to identify the modules whose activity is associated with NPD status in each of the cohorts. Only the modules associated with each NPD were selected for further analysis.

Hub genes in each of the significantly associated modules were identified using the adjacency score in the *CEMiTool* R package. Module overrepresentation analysis (ORA) was performed based on the hypergeometric test, using the C2 Curated KEGG gene set list from the Molecular Signatures Database, version 7 (http://www.gsea-msigdb.org/gsea/msigdb/collections.jsp) [74]. Gene sets with an FDR < 0.05 were considered to be significantly overrepresented. To investigate molecular interactions between the genes within each of the NPD-associated modules, we superimposed module-wise co-expression networks onto human protein–protein interaction data downloaded from BioGRID (https://thebiogrid.org/), which makes use of peer-reviewed publications to manually curate gene and protein interaction data [75].

## Results

### Demographic and clinical information

Demographic and clinical variables for each diagnostic cohort are presented in Table 1. No significant differences in age between cases and controls in any of the diagnostic cohorts were observed. Individuals with PTSD were found to exhibit significantly more childhood trauma compared to trauma-exposed controls (TEC) (p = 0.001). Likewise, a significantly higher number of PTSD cases were found to have been diagnosed with MDD, compared to TECs (p = 0.001). Significantly more schizophrenia cases reported smoking cigarettes compared to controls (p = 0.034).

### Disease-specific gene expression in PTSD, PD and schizophrenia cohorts

Four samples in the PD cohort (one male control, three female patients) and five samples in the schizophrenia cohort [three controls (one male, two female) and two male patients] were identified as outliers, and were therefore omitted from further analyses.

After filtering for genes with low expression (< 10 counts per gene) in each cohort, RNA-seq data for PTSD (n = 11,497 genes), PD (n = 11,763 genes) and SCZ (n = 10,710 genes), were assessed for the degree to which selected variables (e.g., RIN, age, sex, nicotine use, and neutrophil and CD16 monocytes) influenced gene expression and potentially confounded the NPD of interest (PTSD, PD or schizophrenia), using *variancePartition* [68].

The results indicated that, in the PTSD group, the proportion of neutrophils, CD16 monocytes and RIN accounted for the largest contribution to total gene expression variance, whereas MetS status, age, smoking and sex accounted for less of the gene expression variance and affected the expression of relatively fewer genes (Supplementary Figure 1(a) and (b)). Childhood trauma and MDD were not included as variables in the statistical model as they were both significantly associated with PTSD (Table 1) and may thus represent collinear variables.

Violin plots depicting the variance partition analysis in the PD cohort can be viewed in Supplementary Figure 2(a) and (b). Neutrophil and CD16 monocyte proportions, as well as RIN and age, were found to be the main drivers of gene expression variation, with current anti-hypertensive medication use, sex, smoking, metabolic syndrome status and second bead clean-up contributing to the variance in gene expression, although at a lower level.

As with the PD cohort, the top four drivers of gene expression variance in the schizophrenia cohort were found to be neutrophil and CD16 monocyte cell proportions, RIN and age, with sex, smoking, and the second bead clean-up contributing less (Supplementary Figure 3 (a) and (b)).

Only one gene, superoxide dismutase 2 (SOD2) (ENSG00000112096), was found to be differentially expressed between PTSD and TEC at a false discovery rate (FDR) < 0.1 (Supplementary Table 1). No genes were found to be differentially expressed at FDR < 0.1 in either the PD or the schizophrenia cohorts (Supplementary Tables 2 and 3, respectively).

### Weighted gene co-expression network analysis (WGCNA)

Following default gene filtering by *CEMiTool*, 855, 833 and 911 genes were selected for analysis in the PTSD, PD and schizophrenia datasets, respectively. Using *CEMiTool* default settings, a soft-threshold β value of 14 (r^2^ = 0.87) was selected for PTSD, while a soft thresholds of 8 (r^2^ = 0.81) and 5 (r^2^ = 0.81) were selected for PD and schizophrenia, respectively.

**Fig. 1:**
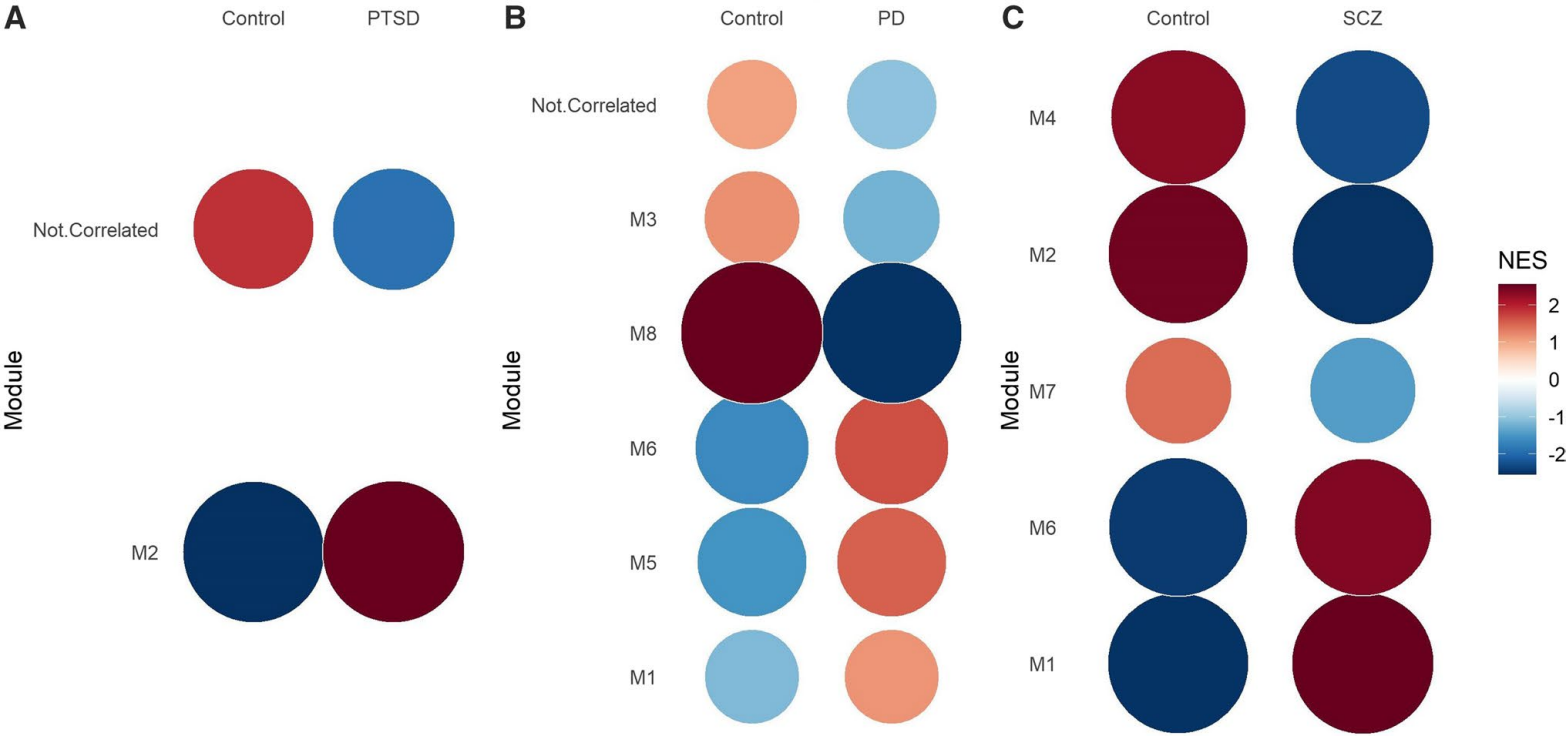
Gene set enrichment analysis showing the module activity for patient and control groups in the (**A**) PTSD, (**B**) PD and (**C**) schizophrenia (SCZ) cohorts. **A** Module 2 genes (n = 158) were found to have significantly increased expression in patients compared to controls in the PTSD cohort. **B** Genes in modules 1 (n = 222), 5 (n = 61), and 6 (n = 60) were found to be significantly increased in PD patients compared to controls, whereas those in modules 3 (n = 122) and 8 (n = 39) were significantly under-expressed in PD patients compared to controls. **C** Genes in modules 1 and 6 were found to be significantly increased in schizophrenia patients compared to schizophrenia controls, whereas those in modules 2, 4 and 7 were significantly under-expressed in schizophrenia patients compared to controls. M, module; SCZ, schizophrenia

For the PTSD dataset, five modules were defined, ranging in size from 187 (Module 1) to 34 (Module 5) genes. Three hundred and twenty-five genes were defined as “*not correlated*”, i.e., they were not placed in any module (Supplementary Table 4). Module 2 (n = 158) was the only module found to be associated with PTSD (p = 3.26 × 10^–16^), following GSEA analysis (Fig. 1(a); Supplementary Table 4).

Eight modules were identified in the PD cohort, ranging in size from 222 to 39 genes (Supplementary Table 5). Five of these modules were found to be significantly differentially expressed between PD patients and controls (Module 1 [n = 222; p < 0.001], Module 5 [n = 61; p < 0.001], Module 6 [n = 60; p < 0.001] and Module 8 [n = 39; p < 0.001] and Module 3 [n = 122; p = 0.001]) (Fig. 1 (b) and Supplementary Table 5).

We identified seven modules, ranging in size from 376 to 49 genes, in the schizophrenia cohort (Supplementary Table 6), and five of these were significantly associated with schizophrenia (Module 1 [n = 376, p = 6.45 × 10^−45^]; Module 2 [n = 185, p = 8.951 × 10^−38^]; Module 4 [n = 79, p = 5.80 × 10^−21^]; Module 6 [n = 63, p = 1.677 × 10^−18^] and Module 7 [n = 49, p = 8.006 × 10^−5^]) (Fig. 1(c) and Supplementary Table 6).

The normalised enrichment score (NES) represents the enrichment score for a module in each group, normalised by the number of genes in the module. The size and colour intensity of the circles correspond to the NES (Supplementary Tables 7–9).

### Pathway enrichment analysis

**Fig. 2:**
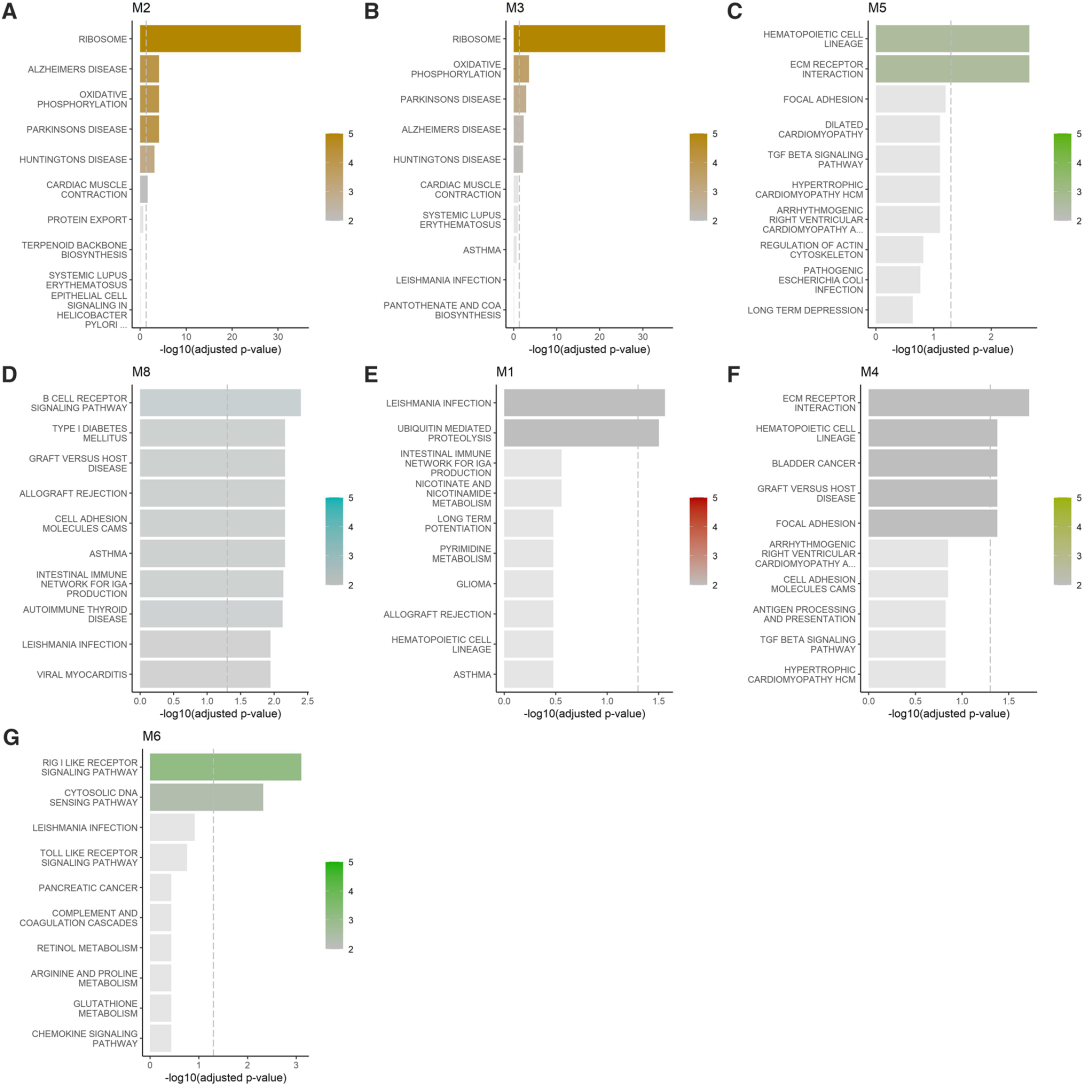
Over representation analysis for (**A**) PTSD, **B**–**D** PD and **E**–**G** schizophrenia. Bar graphs show the − log10 adjusted p-value of the enrichment between genes in modules and KEGG gene sets from MSigDB, the vertical dashed line indicates an adjusted p-value of 0.05. The colour intensities of the bars are proportional to the adjusted p-value

Overrepresentation analysis (ORA) of Module 2 genes in the PTSD cohort identified several enriched KEGG pathways, including *Ribosome* (p = 1.09 × 10^–35^), *Parkinson’s Disease* (p = 7.72 × 10^−5^), *Oxidative phosphorylation* (p = 7.72 × 10^−5^), *Alzheimer’s Disease* (p = 7.72 × 10^−5^), *Huntington’s Disease* (p = 8.40 × 10^−3^) and *Cardiac muscle contraction* pathways (p = 0.024) (Supplementary Table 10, Fig. 2(a)).

Overrepresented pathways were identified in three PD-associated gene modules (modules 3, 5 and 8) (Fig. 2B−D, Supplementary Table 11). The overrepresented KEGG pathways included *Ribosome* (p < 0.001), *Oxidative phosphorylation* (p < 0.001), *Parkinson’s Disease* (p = 0.001), *Alzheimer’s Disease* (p = 0.005) and *Huntington’s Disease* (p = 0.008) pathways in Module 3; *ECM receptor interaction* (p = 0.002), *Hematopoietic cell lineage* (p = 0.002) pathways in Module 5; and *B-cell receptor signalling* (p = 0.004), *Asthma* (p = 0.007), *Cell adhesion molecules* (p = 0.007), *Allograft rejection* (p = 0.007), *Graft-versus-host disease* (p = 0.007), *Type I Diabetes mellitus* (p = 0.007), *Intestinal immune network for IgA production* (p = 0.008), *Autoimmune thyroid disease* (p = 0.008), *Viral myocarditis* (p = 0.01), *Leishmania infection* (p = 0.012), *Hematopoietic cell lineage* (p = 0.014), *Antigen processing and presentation* (p = 0.014) and *Systemic lupus erythematosus* (p = 0.031) pathways (Module 8) (Supplementary Table 11).

Overrepresentation analysis of genes in schizophrenia-associated modules identified a number of enriched KEGG pathways in Module 1 (*Leishmania infection* (p = 0.030), *Ubiquitin mediated proteolysis* (p = 0.033)), Module 4 (*ECM receptor interaction* (p = 0.020), *Focal adhesion* (p = 0.044), *Graft versus host disease* (p = 0.044); *Hematopoietic cell lineage* (p = 0.044), *Bladder cancer* (p = 0.044)) and Module 6 (*RIG I like receptor signalling pathway* (p = 0.0008) and *Cytosolic DNA sensing* (p = 0.005)) (Fig. 2E−G, Supplementary Table 12). No KEGG pathways in modules 2 or 7 were found to be associated with schizophrenia.

### Gene interaction analysis

**Fig. 3:**
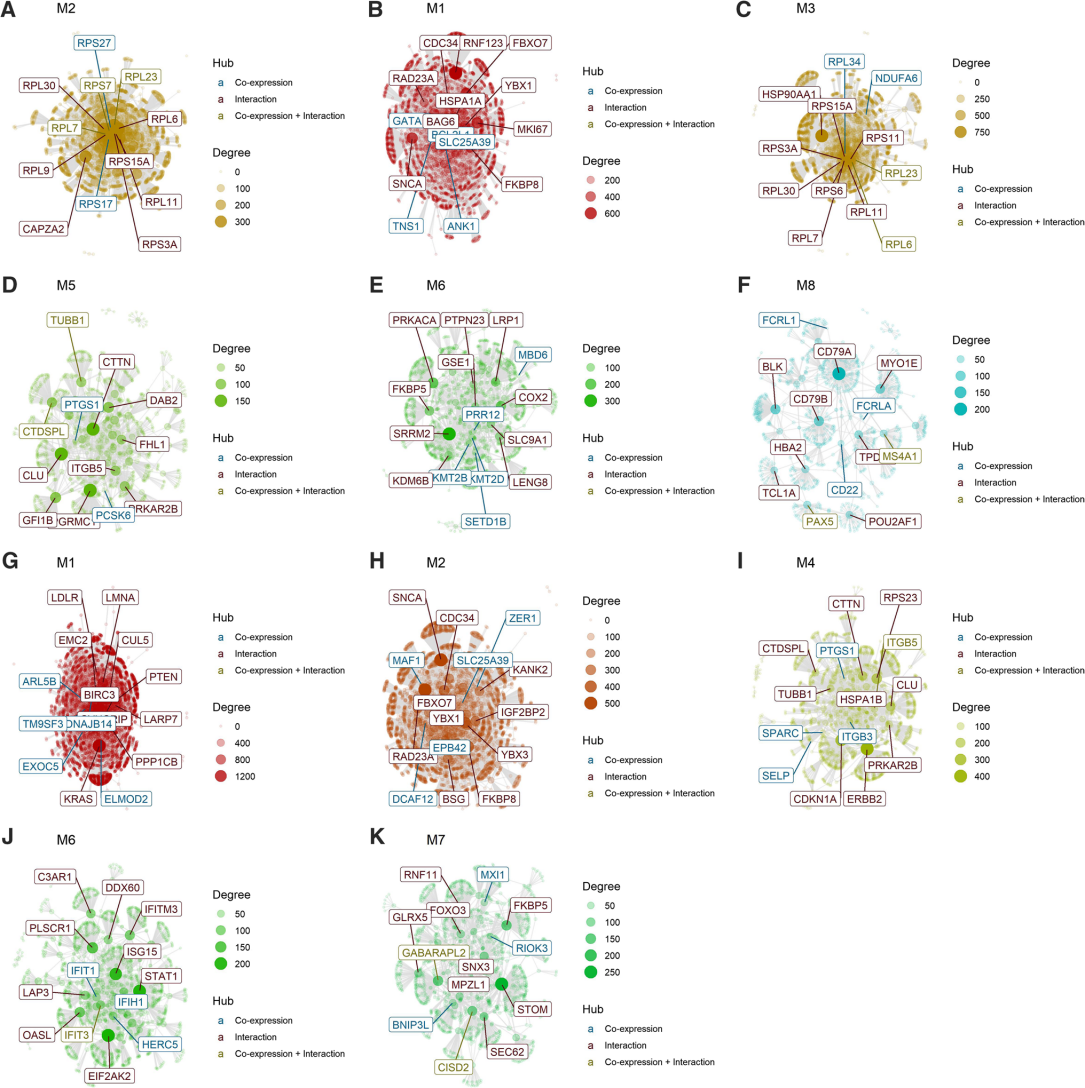
Interaction plots for each of the disorder-associated modules, indicating hub genes in each module. **A** Interaction plot for PTSD-associated Module 2, **B–F** Interaction plots for PD-associated modules 1, 3, 5, 6 and 8, and **G–K** Interaction plots for schizophrenia-associated modules 1, 2, 4, 6 and 7. The hubs are highlighted. The nodes represent the highly co-expressed genes within each module, in addition to those added by protein–protein interaction information, obtained from the BioGRID website. The genes are connected by co-expression and/or protein–protein interaction. Hubs are labelled and coloured based on their “origin”: if they are present in the *CEMiTool* module, they are coloured blue; if they have been inserted from the BioGRID interaction data, they are coloured red, and if they are shared between co-expressed and interaction databases, they are coloured yellow. The degree (connection strength) is reflected by the size of the node

Hubs are defined as nodes (genes) with high connectivity (i.e., high number of neighbours) in the network. These represent nodes that could play an active role in the given context. Gene interaction analysis within PTSD-associated Module 2 identified 11 hub genes (*RPS27*, *RPL6*, *RPL30*, *RPL11*, *RPL7*, *CAPZA2*, *RPL23*, *RPL9*, *RPS3A*, *RPS15*, *RPS7* and *RPS17*) (Fig. 3A). Likewise, a number of gene interactions were identified in the PD-associated modules (Fig. 3B–F) and schizophrenia-associated modules (Fig. 3G–K).

## Discussion

This is the first study to investigate the potential shared mechanisms underlying three common NPDs: PTSD, schizophrenia and PD. It is also, to the best of our knowledge, the first to present results from transcriptomic analyses on each of these NPDs in a Southern African population. Our main aim was to identify pathways underlying the disorders separately, and in combination, using a WGCNA approach.

We identified multiple common KEGG pathway signatures across PTSD Module 2 and PD Module 3, including the KEGG “*Ribosome*”, “*Oxidative phosphorylation*”, “*Parkinson’s Disease*”, “*Huntington’s Disease*” and “*Alzheimer’s Disease*” pathways. Ribosomes are subcellular organelles involved in the synthesis of proteins and comprise two subunits. The large subunit and smaller subunit (60S and 40S subunits, respectively, in eukaryotes) are encoded by ribosomal protein large (RPL) and ribosomal protein small (RPS) genes. The two subunits together form the 80S ribosomal nucleoprotein complex. Ribosomal proteins (RPs) are highly conserved and play an important role in the translation of proteins, specifically, it is thought, in the elongation step of protein synthesis, although the precise function is currently uncertain [76]. Translational homeostasis can be disturbed by alterations in translation initiation rates or RPs themselves, due to factors such as mutations or a decreased or increased number of normal ribosomes (altered gene expression or copy number). Disturbance in translational homeostasis can be pathologically significant in neurodegenerative disorders that are characterised by protein aggregation, one of the hallmark features of PD. Indeed, altered protein synthesis machinery has been found to be associated with alpha-synuclein oligomers in PD, although the expression of RPs was found to be stage- and region-dependent [77]. Interestingly, the downregulation of genes encoding RPs in peripheral tissue has recently been associated with aging [78], the main risk factor for PD [79], pointing towards the possibility of age-related translational dysfunction playing a role in susceptibility to PD. Although the literature on RPs and their association with PTSD is scarce, they have been found to play a role in other psychiatric disorders, including depression and obsessive–compulsive disorder (OCD) [80, 81]. However, the direction of this relationship, i.e., whether increased or decreased translational activity acts as a risk factor, may be disorder-specific.

Our observation that the ribosome pathway is dysregulated in blood in both PTSD and PD may point to dysfunctional protein homeostasis operating on a more systemic level. On the other hand, RPS3, a ribosomal protein that acts as a molecular switch for cell survival and death, has been found to be capable of traversing the blood–brain barrier, where it protected dopaminergic neurons in the substantia nigra from oxidative stress in a 1-methyl-4-phenyl-1,2,3,6-tetrahydropyridine (MPTP) mouse model. This was achieved by upregulating superoxide dismutase 1 (SOD1), which in turn scavenged the reactive oxygen species (ROS), preventing lipid peroxidation [82]. This indicates that alterations of RPs in the blood could affect CNS functioning.

It is important to note that, apart from their crucial role in protein synthesis, RPs also have numerous ribosome-independent functions, including immune signalling, tumorigenesis and development [83, 84]. To this end, RPs have been found to play a role in the interferon-gamma-mediated inflammatory response by modulating gene expression. For example, RPL13-A has been found to resolve inflammatory responses induced by IFN-gamma [83, 84] and RPS3 has been found to regulate genes that are involved in the NFKB immune response, as well as playing a role in cell survival and proliferation [85–87]. It is therefore plausible that altered ribosomal gene expression may result in dysregulation of immune pathways, and possibly contribute to the development of PTSD and/or PD in this way. Although the same pathways may be associated with both disorders, it is not possible to determine whether the same mechanisms within the pathway are disrupted in the disorders. It is, however, interesting that 50% of the PTSD Module 2 and PD Module 3 hub genes overlap, indicating that that some of the mechanisms may be similar.

Interestingly, *RPL6*, a hub gene in both PTSD Module 2 and PD Module 3, has been found to be downregulated in the peripheral blood mononucleocytes in PD patients [88, 89], and has been found to be associated with early-onset PD [90]. Anirudhan et al. [89] observed that RPL6 served as an intermediary, connecting three metalloprotein hubs associated with PD, and that the expression of RPL6 was regulated by serum levels of zinc and magnesium, which is notable given the evidence that metal exposure is an important risk factor for PD [91]. Although the data indicating an association between levels of metals and PTSD is currently sparse, zinc has been found to be associated with both depression and anxiety [92, 93], and serum levels of zinc have been found to be controlled through effective antidepressant pharmacotherapy [94]. In addition, an animal model of PTSD recently showed alterations in zinc concentration in selected brain regions of animals exhibiting a PTSD-like phenotype [95]. Magnesium has been found to play a role in depression, with an inverse relationship noted between dietary magnesium intake and depressive [96] and anxiety [97] symptoms, and the observation that lower serum levels of magnesium were correlated with increased depression as measured using the Patient Health Questionnaire [98]. Although the evidence for the role of magnesium in PTSD is, as with zinc, scarce, the aforementioned findings lay groundwork for further investigation into these relationships.

Genes comprising KEGG pathways of *Focal adhesion* and *ECM receptor interaction* were found to be enriched in the PD Module 5 but under-expressed in the schizophrenia Module 4. Both pathways have been implicated previously in schizophrenia [99–101] and PD [102, 103]. Focal adhesions are specialised regions in the cell where it interacts with the extracellular matrix (ECM). Interactions between cells and the ECM, mediated via transmembrane receptors such as integrins, facilitate the regulation of adhesion, migration and differentiation of cells. This is important in the maintenance of stable neuronal connectivity and the regulation of synaptic plasticity [104]. The involvement of the focal adhesion and ECM receptor interaction pathways in both PD and schizophrenia may point to the aberrant migration of neural progenitor cells during brain development in individuals who suffer from the schizophrenia, and dysregulation of adult synaptic plasticity and neuronal connectivity in PD.

We observed enrichment for several immune system related pathways in modules associated with PD, particularly in Module 8 (*B-cell receptor signalling*, *Asthma*, *Allograft rejection*, *Graft vs host disease*, *Intestinal immune network for IgA production*, *Autoimmune thyroid disease*, *Antigen processing and presentation* and *Systemic lupus erythematosus* KEGG pathways). Prior evidence indicates that chronic low-level inflammation, in both the nervous system and periphery, is a pathophysiological feature of PD [105], and increased cytokine levels observed in the clinical stages of disease suggest the involvement of peripheral inflammation in PD progression [106].

An altered gut microbiome has been found to be associated with PD [107, 108]. It is interesting to note that the ‘*Intestinal immune network for IgA production*’ pathway was found to be dysregulated in the PD cohort. The leucine-rich repeat kinase 2 (*LRRK2*) gene, which is one of the most frequent causes of familial PD [109, 110], is expressed in enteric neurons, where it has been found to modulate the IgA alterations via regulation of neuronal peptides [111]. IgA regulates commensal bacteria via a range of mechanisms, including enhancing bacterial-mucus interactions that aid in establishing and reinforcing the gut mucosal barrier, and suppressing bacterial species that stimulate the immune system and express genes that elicit an inflammatory response (reviewed in [112]). Thus, altered IgA-related gene expression in PD could lead to both altered microbial composition in favour of proinflammatory species, in turn potentially increasing peripheral inflammation, and could reduce the efficacy of the barrier to bacterial translocation. Disease-associated differences in IgA production could, therefore, provide a mechanism underlying recently published findings of microbiome differences in PD [113].

The KEGG *ubiquitin mediated proteolysis* pathway was found to be enriched in Module 1 of the schizophrenia cohort. The ubiquitin proteasome system (UPS) is a complex system that has important roles in diverse processes, including protein labelling for degradation, vesicle and protein transport, membrane receptor recycling, and modulation of cellular responses to inflammation and oxidative stress [114, 115]. Importantly, the UPS has also been described as a crucial regulator of neural development and the maintenance of brain structure and function [116], and has been found to be associated with schizophrenia at a transcriptomic and proteomic level, in both peripheral and central tissue [117–124]. In addition, ubiquitin-conjugating enzyme (UBE2K) (a hub gene of schizophrenia Module 1 *ubiquitin-mediated proteolysis* pathway) has been found to be associated with positive symptom domains of psychosis [121]. Dysregulation of the proteasome system could result in accumulation of dysfunctional and/or damaged proteins. To this end, recent evidence showed that accumulation of ubiquitinated proteins has been detected in a subgroup of schizophrenia patients [125, 126], and it is thought that this may characterise a biological subtype of schizophrenia [124]. This finding represents an interesting point of departure for the current study and should be further investigated in a larger schizophrenia cohort.

No differentially expressed genes were identified in either the PD or the schizophrenia cohorts, and only one gene (superoxide dismutase 2, SOD2) was identified as being differentially expressed between patients and TECs in the PTSD cohort. SOD2, also known as manganese-dependent SOD (MnSOD), is an enzyme that plays an important role as a first line of defence against ROS, and in doing so, protects mitochondria from oxidative damage. ROS perform numerous vital functions in the cell, and especially in the CNS, where they play an important role in normal brain physiology [127]. In healthy individuals, the production of ROS is tightly regulated by, amongst others, an enzymatic defence mechanism, facilitated by antioxidant enzymes such as the SOD family of enzymes, catalase and glutathione peroxidase [128]. Oxidative stress (OXS) is a result of an imbalance in ROS and antioxidant mechanisms responsible for “mopping” the ROS up, and results in detrimental effects, including damage to proteins, lipids and DNA, aberrant downstream signalling, and the stimulation of apoptosis [129–132]. OXS has been associated with neurodegenerative disorders, accelerated cellular aging, vascular disorders, cancers and numerous neuropsychiatric disorders [127, 133]. Recent evidence points towards the association between OXS and PTSD, with animal and human studies implicating increased levels of ROS and OXS in the development of the disorder [127, 134, 135]. The SOD enzymes have been found to be associated with PTSD, although not all results have been consistent, which may reflect differences in diagnostic criteria and research aims. Tezcan et al. [136] observed a positive correlation between total SOD levels in hemolysates and PTSD CAPS scores. On the other hand, Borovac Štefanović et al. [137] observed reduced SOD levels in PTSD compared to healthy controls, and Zieker et al. [138] observed reduced expression of SOD1 in PTSD compared to control individuals. Although the results from the present study require replication in a larger sample, the increased SOD2 expression observed in the present study may reflect increased cytokine production in PTSD patients, and an attempt to protect the cells from OXS, since increased cytokine production has been found to result in the induction of nitric oxide and ROS [139, 140].

The current results need to be viewed in the context of some important limitations. The use of blood instead of brain tissue, although unavoidable in our setting, is a limitation. Blood tissue is complex, comprising a number of subsets of cell populations, each with their own gene expression profile. However, using cell type deconvolution methods, we did not observe significant differences in cell type proportions between cases and controls within each diagnostic cohort. In addition, blood and brain gene expression profiles have been found to correlate, although somewhat variably, in different studies [141–146]. PTSD, schizophrenia, and PD have also been found to possess underlying immunological disturbances, which highlights the value and practicality of using blood samples in gene expression studies for these disorders. Two of the three disorders investigated, PD and schizophrenia, had very small sample sizes, resulting in a lack of statistical power, which may have prevented identification of more subtle gene expression signatures. Although the pathway-based approach provides a broader overview of biological mechanisms, the pathways implicated are intricate, complex and composed of multiple different genes. Further mechanistic studies are required to identify the key components in these pathways, their individual and interactive effects on the molecular level, and their influence on disorder phenotype. Integration of the current RNAseq data with additional -omics investigations, such as DNA methylation and proteomic investigations would have shed light on potential key players in the associated pathways. DNA methylation data are in the process of being analyzed for the PTSD cohort and will be included in a future manuscript.

This study also has several important strengths. Transcriptomic studies provide a measure of functional data that is not afforded by other genetic investigations, such as GWAS. By identifying shared and distinct gene expression profiles associated with these three disorders, our study is aligned with the movement seeking to identify transdiagnostic mechanisms that can bridge the previously siloed investigations of NPDs. Indeed, our results not only point to the complex and multicausal nature of NPDs, but also offer insight into key mechanisms that could be targeted to produce pleiotropic effects on neurobiology and behaviour. This study was also conducted in an African ancestry population. This is relevant given calls to increase the diversity of representation in genetic studies in general, and psychiatric genetic studies, in particular. Furthermore, in keeping with the gene-environment framework, local contexts can influence the risk and course of disorders. For this reason, the results of locally conducted studies are particularly relevant.

In conclusion, we adopted a pathway-based approach to identify overarching mechanisms shared by PTSD, schizophrenia and PD. Our analyses implicated widely acting biological processes influencing ribosome machinery, inflammation, and ubiquitination, all of which have broad scope to affect behaviour. While our data provide preliminary evidence for shared molecular aetiology between PTSD and PD, and between PD and schizophrenia, the results require replication in a larger sample.

## Supplementary Information

Below is the link to the electronic supplementary material.

Supplementary file1 (DOCX 529 KB)

Supplementary file2 (XLSX 2860 KB)

## Data Availability

The datasets generated during and/or analysed during the current study are not publicly available due to lack of participant informed consent and ethics approval to deposit the data in a public repository, but are available from the corresponding author on reasonable request.

## Code availability

The code generated during the current study is available from the corresponding author on request.
