# Supplementary material for: RNA-seq analysis of gene expression profiles in posttraumatic stress disorder, Parkinson’s disease and schizophrenia identifies roles for common and distinct biological pathways
Source: Discov Ment Health. 2022 Mar 3;2(1):6. doi: 10.1007/s44192-022-00009-y (PMC10501040; doi:10.1007/s44192-022-00009-y)
Supplement: Supplementary file 1 — Supplementary file1 (DOCX 529 KB) [file 44192_2022_9_MOESM1_ESM.docx]

**SUPPLEMENTARY FIGURES**


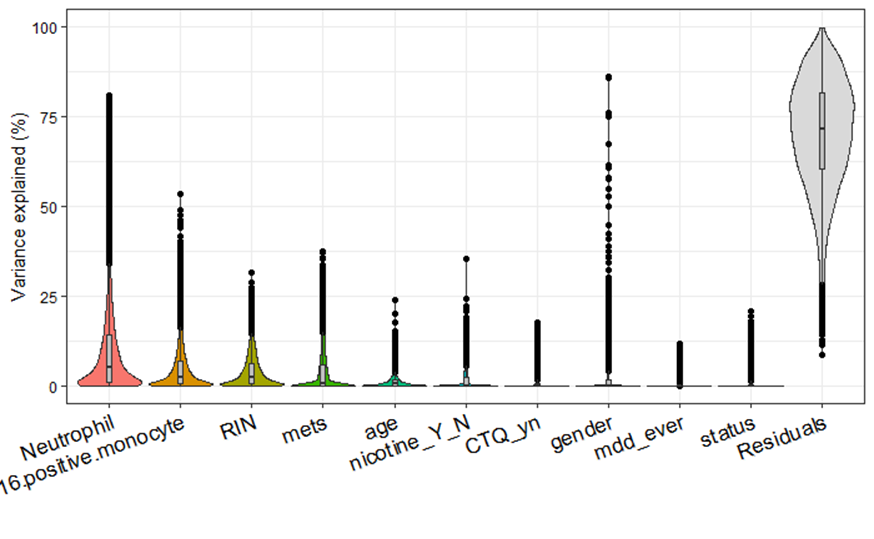


**(a)**


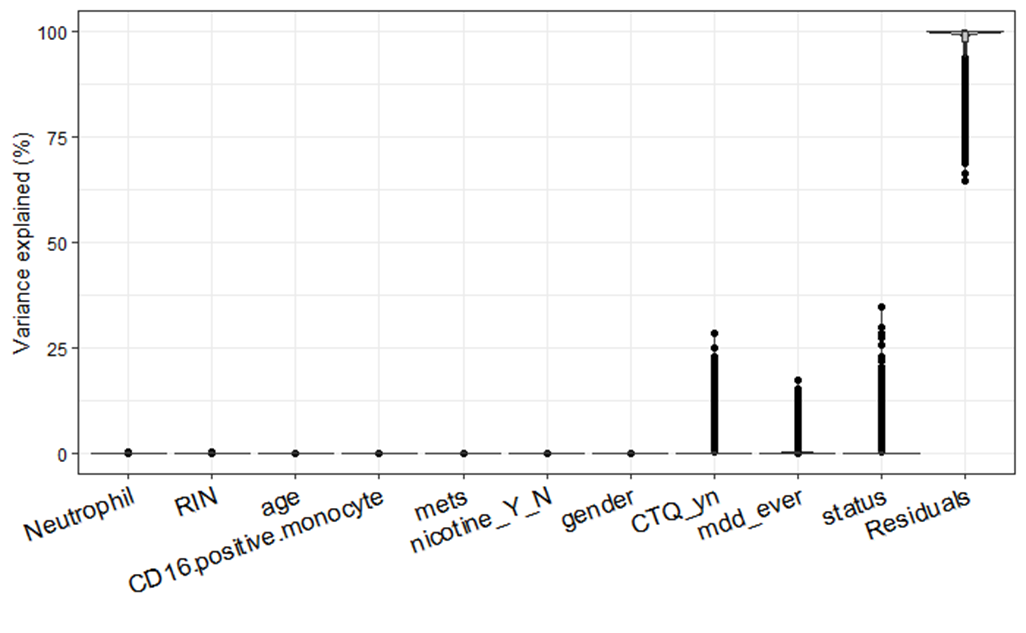


**(b)**

**Supplementary Figure 1 (a)**: Violin plots depicting drivers of variation in gene expression in the PTSD cohort. The *x*-axis represents the selected variables and the *y*-axis indicates the percentage of variance explained by each of these variables. The thickness of each violin bar depicts the proportion of genes corresponding to level of y-axis. The proportions of neutrophil and CD16 monocytes, as well as RIN were the major driver of variation in gene expression in the PTSD cohort. **Figure 1 (b)** shows the variance partition plot after correction for variables Neutrophil proportion, RIN, age, CD16 monocyte proportion, metabolic syndrome status (“*mets*”), smoking status (“*nicotine_Y_N*”), and sex (“*gender*”).


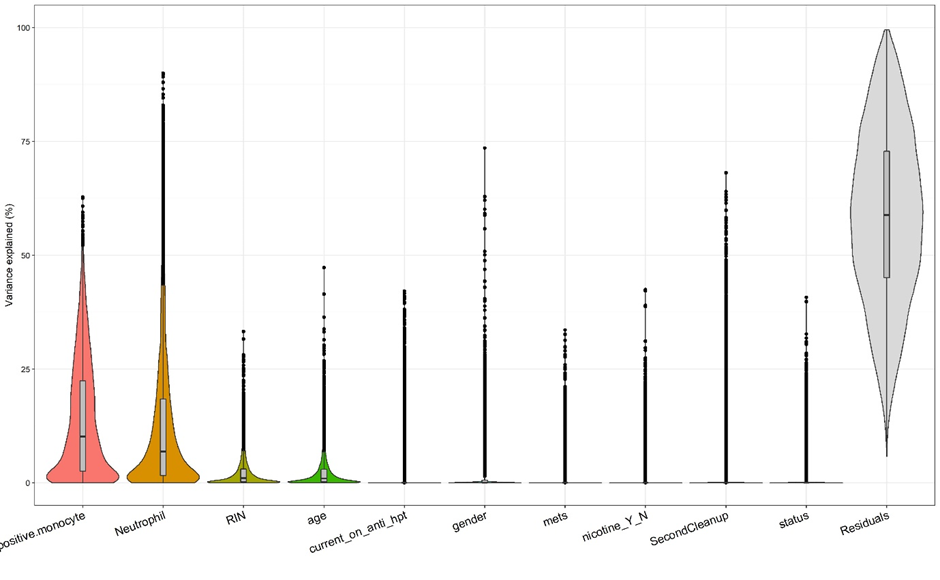


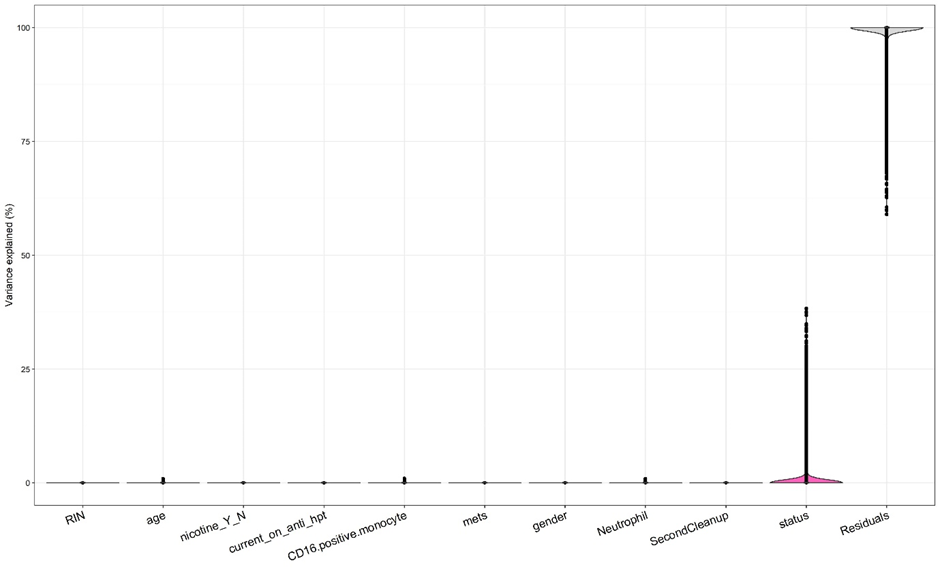


**Supplementary Figure 2 (a):** The proportions of neutrophil and CD16 monocytes, as well as RIN were the major driver of variation in gene expression in the PD cohort. **(b)** shows the variance partition plot after correction for RIN, age, smoking status (“*Nicotine_Y_N*), ant-hypertensive medication (“*current_on_anti_hpt*”), CD16 monocyte proportion, metabolic syndrome status (“*mets*), sex (“*gender*”), neutrophil and additional clean-up procedure (“*SecondCleanup*”).


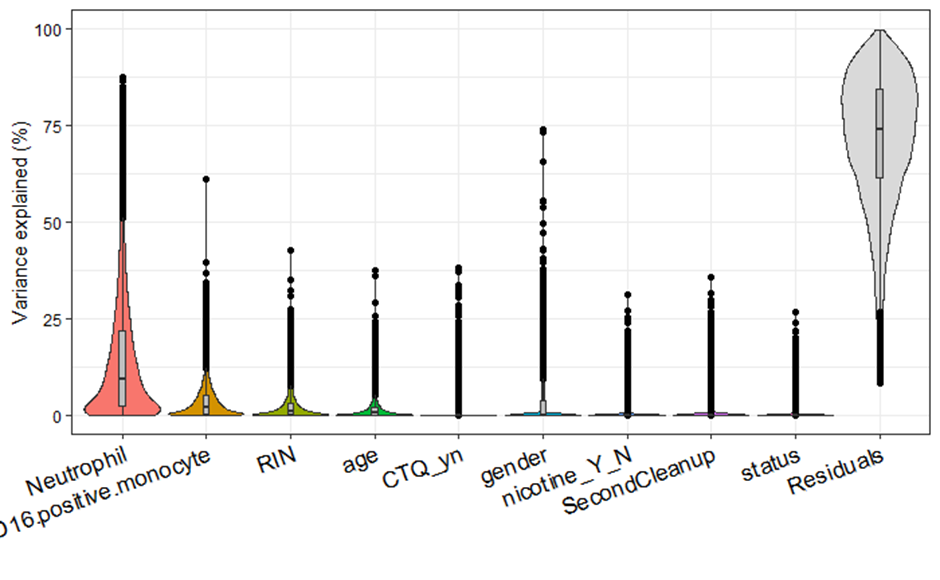


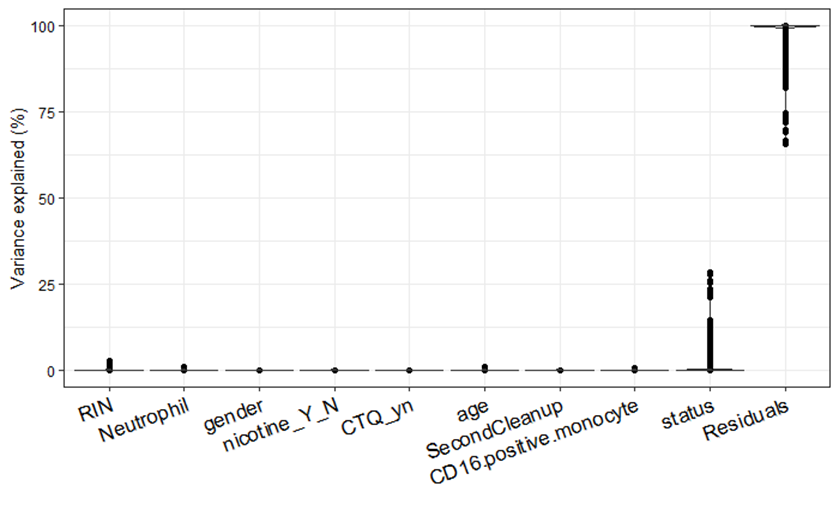


**Supplementary Figure 3 (a):** Violin plots depicting drivers of variation in gene expression in the SCZ cohort. The proportions of neutrophil and CD16 monocytes, as well as RIN were the major driver of variation in gene expression. **(b)** shows the variance partition plot after correction for RIN, Neutrophil proportion, sex (“gender”), smoking (Nicotine_Y_N), childhood trauma (“CTQ_yn”), age, second bead clean-up, and CD16 monocyte proportion.
